# Isolated Posterior Mediastinal Thyroid Nodule Simulating Esophageal Pathology: A Multi-Modality Diagnosis

**DOI:** 10.7759/cureus.26241

**Published:** 2022-06-23

**Authors:** Bushra Riaz, Shayan S Anwar

**Affiliations:** 1 Radiology, Dow University of Health Sciences, Karachi, PAK; 2 Radiology, Aga Khan University Hospital, Karachi, PAK

**Keywords:** multinodular goiter, posterior mediastinal mass, hrct chest (high resolution computed tomography), contrast enhanced ct thorax, barium swallow

## Abstract

Posterior mediastinal goiter is not a common cause of dysphagia, and symptoms can simulate esophageal malignancy. This case report highlights two critical clinical aspects. First, the patient's symptoms of gradually worsening dysphagia to solids and liquids, odynophagia, and hoarseness of voice secondary to retrosternal thyroid nodule extension can simulate esophageal malignancy. Second, a barium swallow study can effectively rule out esophageal pathology even though more advanced studies, like High-resolution computed tomography (HRCT), are inconclusive. We present a unique case of isolated posterior mediastinal exophytic thyroid nodule simulating the symptoms of esophageal pathology.

## Introduction

Posterior mediastinal goiter constitutes only 10-15% of the intrathoracic goiter. Various symptoms like hoarseness of voice, dysphagia, and difficulty breathing occur due to the mass effect of an enlarged goiter on neighboring organs and tissues [1]. Radiographic imaging is an essential tool in diagnosing posterior mediastinal goiter. The multiple imaging modality approaches help illustrate the association of surrounding structures to the posterior mediastinal mass, as in this case, based on the origin of the mediastinal lesion.

High-resolution computed tomography (HRCT) utilizes thin sections, and images are reconstructed using a sharp algorithm such as a bone algorithm. Although this enhances visible noise, it is a negligible factor in lung imaging due to the lung's high air-to parenchyma ratio. But it makes the mediastinal structures "grainy," and tissues with markedly different attenuations like air and lung tissue are visible with an edge enhancement effect [2], so it is the modality of choice for lung parenchymal disease. 

However, barium swallow is more reliable for mediastinal mass evaluation when symptoms point towards esophageal pathology. Furthermore, to rule out any functional disorder of the pharynx, mucosal diseases of the esophagus, which are seen as mucosal irregularities, and its motility disorders, it certainly has an edge of easy availability and economical as opposed to enhancing CT scan or HRCT. Therefore, barium swallow remains the primary imaging modality in assessing esophageal diseases like dysphagia and motility disorders [3].

Although barium swallow may outline esophageal compression from a mediastinal mass as the cause of symptoms, enhanced CT is the imaging modality of choice to evaluate and characterize a posterior mediastinal mass [4]. With the approach of multidetector CT, volume acquisition CT has become customary to acquire. The edge of getting a volume acquisition CT scan is that it reconstructs the images entirely in three different planes: the axial, sagittal, and coronal planes. Viewing the anatomy and pathology in all three planes is particularly helpful when evaluating the extent of disease in a patient [5].

## Case presentation

A 70-year-old male with no known comorbidities presented to us with complaints of gradual dysphagia to both solids and liquids accompanying odynophagia and hoarseness of voice with intermittent choking. He also had a productive cough for 15 days. The patient's dysphagia was mild to moderate at first but worsened over the past month. There was no history of involuntary weight loss, heartburn or indigestion, chest pain, vomiting, or diarrhea on additional questioning. And no complaints of heat or cold intolerance or tremors. He was a non-smoker and non-alcoholic. On examination, an old-aged man of average height and build, with no apparent swelling in the neck or chest, no halitosis, and no lymph node enlargement found; the rest of the gastrointestinal examination was also unremarkable. General physical and systemic examinations were within usual limits.

**Figure 1:**
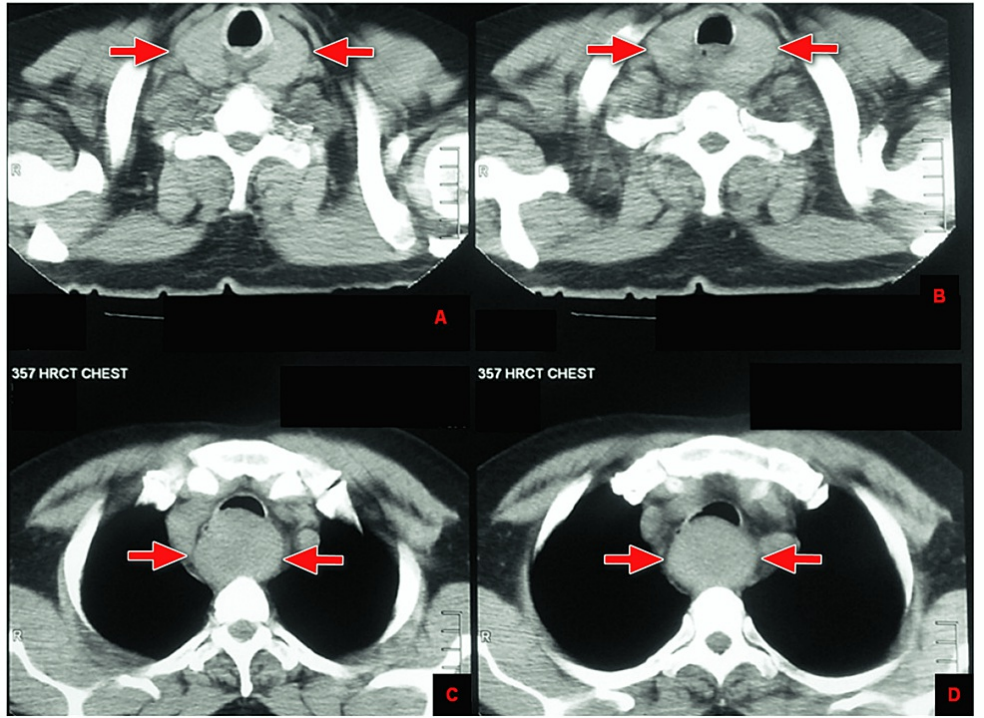
Axial HRCT of the chest with red arrows demonstrating a large smooth, soft tissue mass in the prevertebral location, extending from the cricoid post area (A and B) to the level of the aortic arch (C and D).

As High-resolution CT scan of the chest was already done when the patient presented to us and reported a large mass of smooth and soft tissue identified in the prevertebral location, which extends from the cricoid post area up to the level of the aortic arch, highly suggestive of neoplastic esophageal or mediastinal lesion (Figure 1).

**Figure 2:**
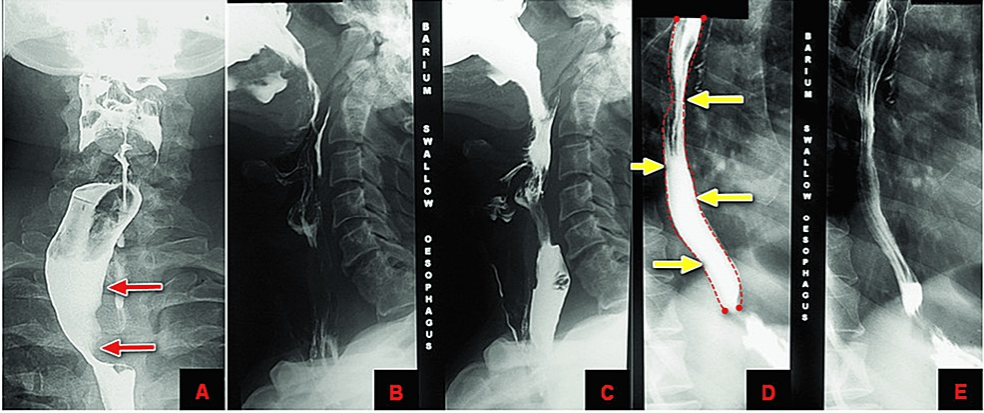
Barium swallow study with red arrows showing a large space-occupying prevertebral lesion identified in the lower cervical and upper dorsal spine, causing anterior and right lateral displacement of the esophagus (A) and causing a smooth posterior extrinsic impression over the posterior wall (B and C) with no mucosal erosion or destruction of the esophagus (Image E) and demonstrated by the dotted line and yellow arrows in D.

So, the Barium swallow study was advised to rule out esophageal pathology, which showed a large space-occupying prevertebral lesion causing anterior and right lateral displacement of the esophagus and causing smooth posterior extrinsic impression over the posterior wall with no mucosal erosion or destruction. In addition, no destructive lesion was identified within the esophagus or hypopharynx (Figure 2).

**Figure 3:**
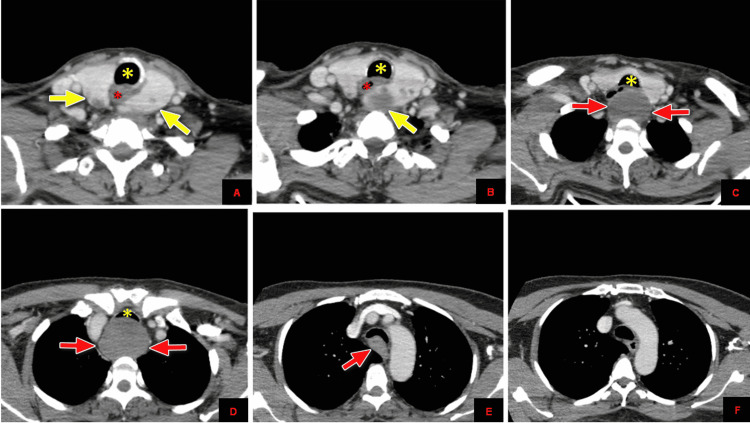
Contrast-enhanced CT scan of the chest with yellow arrows demonstrating that both thyroid glands are enlarged, showing multiple subcentimeter hypoattenuating nodules (A and B). A large exophytic oval-shaped solid lesion is seen arising from the posteroinferior pole of the left thyroid gland (red arrows) in C, which is extending in prevertebral space and markedly compressing and displacing the esophagus (red asterisk) and trachea (yellow asterisk) in D, E and F. It is smoothly marginated with no definite invasion.

**Figure 4:**
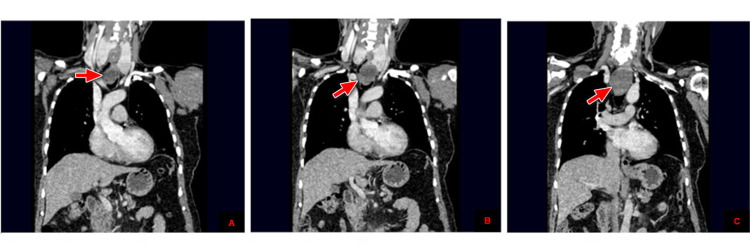
CT scan of the chest with contrast in the coronal plane with red arrows pointing to the thyroid mass in A and B. It extends from T1 to the T3 level, and no calcification or necrosis is seen within it (C).

For further precise lesion characterization, a contrast-enhanced CT scan neck and chest was advised. CT scan revealed that both thyroid glands are enlarged, showing multiple subcentimeter hypoattenuating nodules. A large exophytic inferiorly hanging oval-shaped solid lesion is seen arising from the posteroinferior pole of the left thyroid gland, which is extending in prevertebral space and markedly compressing and displacing the esophagus and trachea. It approximately measures 5.1 x 3.8 x 5.0 cm in craniocaudal, anteroposterior, and transverse diameters, respectively. It extends from T1 to the T3 level, and no calcification or necrosis is seen. It is smoothly marginated and markedly compressing the esophageal lumen with no definite invasion (Figure 3, 4).

Appearances were more in favor of benign thyroid nodules with unusual presentation. No significant cervical lymph nodes were visualized, and no significant mediastinal or hilar lymphadenopathy. The major vessels of the mediastinum appear normal. SERUM TSH, FREE T3, AND T4 were done and within normal limits, as shown in Table 1.

**Table 1:** Thyroid Panel TSH- Thyroid-stimulating hormone.

TESTS	RESULT	REFERENCE RANGE
TSH	1.710 uIU/mL	0.450 - 4.500 uIU/mL
Thyroxine (T4)	5.8 ug/dL	4.5 - 12.0 ug/dL
Thyroxine (T4) Free	1.2 ng/dL	0.82 - 1.77 ng/dL
Triiodothyronine (T3) Free	1.5 pg/mL	2.0 - 4.4 pg/mL

The patient was euthyroid. Based on the symptoms and imaging findings, a diagnosis of secondary intrathoracic goiter was made, and surgical resection of the thyroid gland was done. After surgical resection, patient symptoms remarkably improved within three weeks, mild hoarseness of voice remained for four weeks, but dysphagia, odynophagia, and choking were utterly relieved. Also, follow-up post-surgical biopsy findings were consistent with benign multinodular goiter. The patient's general condition remains good after six months of surgery, with no post-surgical complications seen.

## Discussion

We reported a case of a patient who presented with symptoms (dysphagia, odynophagia, hoarseness of voice, cough, and choking) that pointed towards esophageal, oropharyngeal, mediastinal, or lung mass. Barium swallow was done, which concluded no esophageal or oropharyngeal lesion. HRCT ruled out lung pathology or mass but concluded there was a mediastinal mass. CT SCAN Chest teased out the diagnosis of secondary intrathoracic goiter. The appearance of the nodule, with the euthyroid status of the patient, explained the symptoms and helped reach the diagnosis. Depending on the origin of the mediastinal lesion, all three modalities have their benefits and limitations.

When investigating esophageal pathology, a simple investigation such as barium swallow can prove more effective in ruling out esophageal pathology and narrowing down the diagnosis. Whereas HRCT is best for diagnosing lung parenchymal lesions, an enhanced CT scan is the most important diagnostic method for intrathoracic goiter, particularly the posterior mediastinal goiter situated close to major blood vessels of the thorax and pressing the trachea and esophagus, ascertainment of the correct size and location of the lesion needs multilayer images of the mediastinum done by post-contrast CT scan [6]. This discussion will describe some key features of intrathoracic goiter, its types, and the treatment.

The term intrathoracic goiter refers to a goiter with most of its mass located in the mediastinum. The primary location of the intrathoracic goiter is the anterior mediastinum, and the posterior mediastinal goiter constitutes only 10-15% of the intrathoracic goiter [7]. It is classified into primary and secondary intrathoracic goiter based on the source of origin [8].

Primary intrathoracic goiter is an ectopic thyroid tissue separated from a cervical thyroid mass and infrequent (1%). In contrast, secondary intrathoracic goiter is a part of the retrosternal thyroid goiter, as seen in our case. The recommended treatment for secondary intrathoracic goiter is thyroidectomy, either total or partial, succeeding levothyroxine replacement [9].

Compression of the airway due to the increasing mass of goiter is the primary culprit of respiratory symptoms [10]. Therefore, numerous oropharyngeal and respiratory symptoms like hoarseness of voice, odynophagia, dysphagia, and difficulty breathing occur due to pressure on the surrounding mediastinal structures because of the size of the enlarged goiter; surgical resection of the thyroid is recommended [11].

Hypoparathyroidism, recurrent laryngeal nerve palsy, and hemorrhage are the most common complications of thyroidectomy, although their incidence is low. The most common of these complications is hypoparathyroidism, with an incidence rate between 0.5 and 65% [12]. The most common iatrogenic cause of vocal cord paralysis is thyroid surgery among head and neck surgery. Identification of the recurrent laryngeal nerves and careful surgical technique can significantly decrease the incidence of this complication [13].

## Conclusions

The unusual location of thyroid mass, as in this patient, retrosternal and posterior mediastinum, can cause compression of the esophagus and trachea, leading to presenting symptoms similar to the esophageal lesion. When investigating esophageal pathology, a simple investigation such as barium swallow can be more effective in ruling out esophageal pathology, narrowing down the diagnosis, saving time, and preventing unnecessary invasive tests like an esophageal biopsy. An enhanced CT scan can help reach the diagnosis without delay.

A radiographic investigation like an enhanced CT scan is the most important diagnostic method for intrathoracic goiter. In cases like this, after the CT scan teases out the diagnosis, the intrathoracic goiter causing compression symptoms should be surgically resected as soon as possible to avoid emergency complications such as acute respiratory distress.
